# Impaired neuropathic pain and preserved acute pain in rats overexpressing voltage-gated potassium channel subunit Kv1.2 in primary afferent neurons

**DOI:** 10.1186/1744-8069-10-8

**Published:** 2014-01-29

**Authors:** Longchang Fan, Xiaowei Guan, Wei Wang, Jian-Yuan Zhao, Hongkang Zhang, Vinod Tiwari, Paul N Hoffman, Min Li, Yuan-Xiang Tao

**Affiliations:** 1 Department of Anesthesiology and Critical Care Medicine, Johns Hopkins University School of Medicine, Baltimore, MD 21205, USA; 2 Department of Anesthesiology, Tongji Hospital, Tongji Medical College, Huazhong University of Science and Technology, Wuhan 430030, PR China; 3 The Solomon H. Snyder Department of Neuroscience, Johns Hopkins University School of Medicine, Baltimore, MD 21205, USA; 4 Department of Ophthalmology, Johns Hopkins University School of Medicine, Baltimore, MD 21205, USA; 5 Department of Anesthesiology, New Jersey Medical School, Rutgers, The State University of New Jersey, 185 S. Orange Ave., MSB, F-548, Newark, NJ 07103, USA

**Keywords:** Potassium channels, Kv1.2, Distribution, Dorsal root ganglion, Neuropathic pain

## Abstract

Voltage-gated potassium (Kv) channels are critical in controlling neuronal excitability and are involved in the induction of neuropathic pain. Therefore, Kv channels might be potential targets for prevention and/or treatment of this disorder. We reported here that a majority of dorsal root ganglion (DRG) neurons were positive for Kv channel alpha subunit Kv1.2. Most of them were large and medium, although there was a variety of sizes. Peripheral nerve injury caused by lumbar (L)_5_ spinal nerve ligation (SNL) produced a time-dependent reduction in the number of Kv1.2-positive neurons in the ipsilateral L_5_ DRG, but not in the contralateral L_5_ DRG. Such reduction was also observed in the ipsilateral L_5_ DRG on day 7 after sciatic nerve axotomy. Rescuing nerve injury-induced reduction of Kv1.2 in the injured L_5_ DRG attenuated the development and maintenance of SNL-induced pain hypersensitivity without affecting acute pain and locomotor function. This effect might be attributed to the prevention of SNL-induced upregulation of endogenous Kv1.2 antisense RNA, in addition to the increase in Kv1.2 protein expression, in the injured DRG. Our findings suggest that Kv1.2 may be a novel potential target for preventing and/or treating neuropathic pain.

## Introduction

Peripheral nerve injury often causes persistent neuropathic pain that is characterized by spontaneous ongoing or intermittent burning pain, allodynia, and hyperalgesia [1]. Treatment options are limited in part because our understanding of the mechanisms that underlie the induction and maintenance of neuropathic pain is incomplete [2,3]. Peripheral nerve injury causes several changes in the nervous system, including abnormal ectopic firing from neuromas and dorsal root ganglion (DRG) neurons [1,4,5]. These abnormal neuronal activities play a critical role in the development and maintenance of neuropathic pain.

DRG neurons express a variety of ion channels, including voltage-gated potassium (Kv) channels [6-8]. Kv channels are tetramers of superfamily-specific channel subunits that comprise ion-conducting integral protein α subunits and auxiliary cytoplasmic β subunits. More than a dozen α subunits of the Kv channel have been isolated from mammalian cells and divided into 12 subfamilies, Kv1–12; many subfamilies consist of more than one subunit [9,10]. Because Kv channels are critical for establishing resting membrane potential and controlling neuronal excitability [11], changes in the expression levels and operating characteristics of Kv channels in the DRG after peripheral nerve injury may contribute to abnormal activities of DRG neurons in neuropathic pain.

The Kv1.2 subunit may participate in the formation of Kv channel tetramers in most DRG neurons. The mRNA for Kv1.1 and Kv1.2 is highly abundant, whereas that of Kv1.3, Kv1.4, Kv1.5, and Kv1.6 is present at lower levels in the DRG [12]. Some controversy exists regarding the subpopulation distribution of Kv1.2 in rat DRG. For example, Ishikawa et al. [13] showed that Kv1.2 was expressed in small DRG neurons, whereas Rasband et al. [14] revealed that Kv1.2 was predominantly distributed in large DRG neurons. Interestingly, peripheral nerve injury down-regulated the expression of Kv1.2 in the injured DRG [12-15]. This down-regulation may be responsible for the nerve injury-induced increase in the ectopic discharge activity observed in DRG neurons [16]. Therefore, DRG Kv.1.2 might be a potential target for neuropathic pain treatment.

In the present study, we first characterized the subpopulation distribution patterns and neurochemical properties of Kv1.2-positive cells. Second, we used immunohistochemistry to further examine the temporal change in the number of K v1.2-positive neurons in the injured DRG in two peripheral nerve injury models: lumbar (L)_5_ spinal nerve ligation (SNL) and sciatic nerve transection (axotomy). Finally, we tested whether rescuing Kv1.2 downregulation by over-expressing Kv1.2 RNA in the injured DRG affected SNL-induced neuropathic pain, acute pain, and locomotor functions.

## Materials and methods

### Animals

Male Sprague–Dawley rats (250–300 g, Harlan Bioproducts for Science, Indianapolis, IN) were housed in cages on a standard 12:12 h light/dark cycle. Water and food were available *ad libitum* until rats were transported to the laboratory approximately 1 h before experiments. All experimental procedures received prior approval from the Animal Care and Use Committee at the Johns Hopkins University. Animal procedures were consistent with the ethical guidelines of the National Institutes of Health and the International Association for the Study of Pain and the ethical guidelines to investigate experimental pain in a conscious animal. Efforts were made to minimize animal suffering and to reduce the number of animals used. The experimenters were blind to the treatment condition during the behavioral testing.

### DRG microinjection

DRG microinjection was carried out as described [17-19]. Briefly, the DRG was exposed by laminectomy. A midline incision was made in the lower lumbar back region, and the L_5_ vertebral body was exposed. The lamina was then removed with small ronguers. After the DRG was exposed, viral solution (2 μl/DRG, 10^12^ particles/ml) was injected into one site in the L_5_ DRG with a glass micropipette connected to a Hamilton syringe. The pipette was removed 10 min after injection. The surgical field was irrigated with sterile saline and the skin incision closed with wound clips.

### L_5_ SNL-induced neuropathic pain model

L_5_ SNL was carried out according to our previous protocols [20-23]. Briefly, after the rats were anesthetized with isoflurane, the left L_6_ transverse process was removed to expose the L_4_ and L_5_ spinal nerves. The L_5_ spinal nerve was then carefully isolated, tightly ligated with 3–0 silk thread, and transected just distal to the ligature. The surgical procedure for the sham group was identical to that of the SNL group, except that the spinal nerve was not transected or ligated.

### Sciatic nerve axotomy

After the rats were anesthetized with isoflurane, the left sciatic nerve was exposed, and the nerve was cut at a point approximately 1 cm distal to the exit point of spinal nerve roots according to the method described previously [20]. Proximal and distal stumps were separated to ensure full transection. In the sham group, the surgical procedure was identical, except that the sciatic nerve was not transected.

### Capsaicin-induced acute pain

The experimental procedure used for the capsaicin test was carried out as described previously [24]. Briefly, fifty μl of vehicle (0.7% alcohol) or capsaicin (2 μg) was injected intradermally under the dorsal surface of the rat left hindpaw by use of a microsyringe with a 26-gauge needle. The amount of time that animals spent licking and/or lifting the injected paw was measured with a stopwatch and was considered as an indicator of the nocifensive response. The animal was observed individually for 5 min immediately after the injection of vehicle or capsaicin. Mechanical, cold, and thermal tests as described below were carried out 30 min after the injection of vehicle or capsaicin.

### Behavioral testing

Mechanical paw withdrawal thresholds were measured with the up–down testing paradigm 1 day before surgery and on days 3, 7, and 14 after SNL or sham surgery according to our previous reports [20-22]. Briefly, the rat was placed in a Plexiglas chamber on an elevated mesh screen. Von Frey hairs in log increments of force (0.407, 0.692, 1.202, 2.041, 3.63, 5.495, 8.511, 15.14 g) were applied to the plantar surface of the left and right hind paws. The 2.041-g stimulus was applied first. If a positive response occurred, the next smaller von Frey hair was used; if a negative response was observed, the next higher von Frey hair was used. The test was ended when (i) a negative response was obtained with the 15.14-g hair, (ii) four stimuli were applied after the first positive response, or (iii) nine stimuli were applied to one hind paw.

Paw withdrawal latencies to cold were measured with a cold plate, the temperature of which was monitored continuously. A differential thermocouple thermometer (Harvard Apparatus, South Natick, MA) attached to the plate provided temperature precision of 0.1°C. Each animal was placed in a Plexiglas chamber on the cold plate, which was set at 0°C. The length of time between the placement of the hind paw on the plate and the animal jumping, with or without paw licking and flinching, was defined as the paw withdrawal latency. Each trial was repeated three times at 10-min intervals for the paw on the ipsilateral side. A cutoff time of 60 s was used to avoid paw tissue damage.

Paw withdrawal latencies to heat were measured with a Model 336 Analgesia Meter (IITC Life Science Instruments, Woodland Hills, CA, USA). Each animal was placed in a Plexiglas chamber on a glass plate (at 25°C) located above a light box. Radiant heat was applied by aiming a beam of light through a hole in the light box through the glass plate to the middle of the plantar surface of each hind paw. When the animal lifts its paw in response to the heat, the light beam is turned off. The length of time between the start of the light beam and the foot lift was defined as the paw withdrawal latency. Each trial was repeated five times at 5-minute intervals for each paw. A cutoff time of 20 seconds was used to avoid paw tissue damage.

The following locomotor function tests were performed: (1) Placing reflex: The rat was held with the hind limbs slightly lower than the forelimbs, and the dorsal surfaces of the hind paws were brought into contact with the edge of a table. The experimenter recorded whether the hind paws were placed on the table surface reflexively; (2) Grasping reflex: The rat was placed on a wire grid and the experimenter recorded whether the hind paws grasped the wire on contact; (3) Righting reflex: The rat was placed on its back on a flat surface and the experimenter noted whether it immediately assumed the normal upright position. Scores for placing, grasping, and righting reflexes were based on counts of each normal reflex exhibited in five trials.

### Plasmid construction, cell culture and transfection, and virus production

To construct the full-length Kv1.2 expression plasmid, total RNA was extracted from rat DRG tissue using Trizol. The reverse transcription was performed with specific primer (5′-GGGTGACTCTCATCTTTGGA-3′). Then the full-length sequence of Kv1.2 cDNA was amplified using primers (Forward: 5′-ATCCACCGGTGCCACCATGACAGTGGCTACC GGAGA-3′; Reverse: 3′-ATAGTTTAGCGGCCGCTCAGACATCAGTTAACATTTTGG-5′). The PCR products were subsequently digested using AgeI and NotI and cloned into the restriction sites of the proviral plasmids (pHpa-trs-SK, provided by Dr. R.J. Samulski, University of North Carolina, Chapel Hill) to replace enhanced GFP (EGFP) sequence. The cDNA sequence and recombinant clones were verified by using DNA sequencing. The resulting two vectors expressed EGFP and Kv1.2 RNA under the control of the cytomegalovirus promoter. AAV5 viral particles carrying the two cDNAs were produced at the University of North Carolina Vector Core.

Human embryonic kidney 293 (HEK293) cells (1 × 10^6^) were seeded in 6-well culture plates and cultured in Dulbecco’s modified Eagle medium supplemented with 10% fetal bovine serum and 1% penicillin/streptomycin solution (10,000 U/ml and 10 mg/ml, respectively). After 24 hours of culture, 2.5 μg of the plasmids (pHpa-trs*-*Kv1.2 or pHpa-trs-EGFP) were transfected into the HEK-293 T cells with Lipofectamine 2000 (Invitrogen, Carlsbad, CA) according to the manufacturer’s instructions. After the 4-day incubation, the cells were collected and subjected to Western blot analysis as described below.

### Total RNA preparation and quantitative real-time RT-PCR

RNA extraction and real-time RT-PCR were performed according to our previously published protocols [20,21]. Briefly, rats were decapitated, and bilateral L_4/5_ DRGs and L_5_ spinal cord were rapidly collected. To obtain enough RNA, L_4_ or L_5_ DRGs from one side of three rats per time point were pooled. Total RNA was extracted with the Trizol method (Invitrogen, Carlsbad, CA), precipitated with isopropanol, treated with RNase-free DNase I (1 μL/20 μL; Promega Corp., Madison, WI), and reverse-transcribed by using the Omniscript kit (Qiagen, Valencia, CA) with specific primers [Kv1.2: 5′-GGG TGA CTC TCA TCT TTG GA-3′, Kv1.2 AS: 5′-CGTCACACCTCCTGAGGACAG-3′, or glyceraldehyde-3-phosphate dehydrogenase (GAPDH, an internal control for normalization): 5′-GAG CAC AGG GTA CTT TAT TGA T -3′]. cDNA was amplified by real-time PCR by using the probes for Kv1.2/Kv1.2 AS (5′-/56-FAM/TGC TGT TGG AAT AGG TGT GGA AGG T/BHQ_1/-3′) (Integrated DNA Technologies, Coralville, IA), and GAPDH [probe/primers were obtained from Applied Biosystems (catalog number: 4331182), Foster City, CA]. The Kv1.2 PCR primer sequences were 5′-AGA AAG GGT CGG TGA AGG AGG T-3′ (forward) and 5′-GTG TGG CTT CTC TTT GAA TAC C-3′ (reverse). Kv1.2 AS PCR primer sequence were 5′-GTG TGG CTT CTC TTT GAA TAC C-3′ (forward) and 5′-AGA AAG GGT CGG TGA AGG AGG T-3′ (reverse). Real-time PCR for each sample was run in quadruplicate in a 20-μL reaction with TaqMan Universal PCR Master Mix (Applied Biosystems). Reactions were performed in an ABI 7500 Fast Real-Time PCR System (Applied Biosystems). The amplification protocol was: 3 min at 95°C, followed by 45 cycles of 10 sec at 95° for denaturation and 45 sec at 58° for annealing and extension. Ratios of ipsilateral-side mRNA levels to contralateral-side mRNA levels were calculated by using the ΔCt method (2^−ΔΔCt^) at a threshold of 0.02. All data were normalized to GAPDH, which has been demonstrated to be stable even during peripheral nerve injury [20,21,25].

### Western blot analysis

The protocol for Western blot analysis has been described previously [26,27]. In brief, cultured cells and bilateral L_5_ DRGs were collected and rapidly frozen in liquid nitrogen. The cultured cells were sonicated and tissues homogenized in chilled lysis buffer (50 mM Tris, 1 mM phenylmethylsulfonyl fluoride, 1 mM EDTA, 1 μM leupeptin). The crude homogenate was centrifuged at 4°C for 15 min at 1,000 × *g*. The supernatant was collected and the pellet (nuclei and debris fraction) discarded. After protein concentration was measured, the samples were heated at 99°C for 5 min and loaded onto a 4% stacking/7.5% separating SDS-polyacrylamide gel (Bio-Rad Laboratories, Hercules, CA). The proteins were then electrophoretically transferred onto a polyvinylidene difluoride membrane (Immobilon-P, Millipore, Billerica, MA). The membranes were blocked with 3% nonfat milk in Tris-buffered saline containing 0.1% Tween-20 for 1 h and incubated with primary mouse anti-Kv1.2 (NeuroMab, Davis, CA), primary mouse anti-Kv1.4 (NeuroMab), or primary mouse anti-β-actin (Santa-Cruz Biotechnology, Santa Cruz, CA) overnight under gentle agitation. β-actin was used as a loading control. The proteins were detected by horseradish peroxidase-conjugated anti-mouse secondary antibody and visualized by chemiluminescence regents (ECL; Amersham Pharmacia Biotech, Piscataway, NJ) and exposure to film. The intensity of blots was quantified with densitometry. The blot density from the contralateral side of the EGFP-treated group was set as 100%. The relative density values from the ipsilateral side of the EGFP group and the ipsilateral and contralateral sides of Kv1.2-treated groups after sham or SNL were determined by dividing the optical density values from these groups by the value of the contralateral side in the EGFP-treated group after they were normalized to the corresponding β-actin.

### Immunohistochemistry

After being deeply anesthetized with isoflurane, the rats were perfused through the ascending aorta with 100 mL of 0.01 M phosphate-buffered saline (PBS, pH 7.4) followed by 400 mL of 4% paraformaldehyde in 0.1 M phosphate buffer (pH 7.4). L_5_ DRGs were removed, post-fixed in the same fixative for 2–4 h, and then cryoprotected in 30% sucrose in 0.1 M phosphate buffer overnight at 4°C. Transverse sections (20 μm thickness) were cut on a cryostat. For single labeling, every fourth section was collected (at least 3–4 sections/DRG). For double labeling, five sets of sections (2–3 sections/set) were collected from each DRG by grouping every fifth serial section.

Single-label immunofluorescence histochemistry was carried out as described previously [28,29]. After being blocked for 1 h at 37°C in PBS containing 10% goat serum and 0.3% TritonX-100, the sections were incubated alone with primary mouse monoclonal anti-Kv1.2 overnight at 4°C. The sections were then incubated with goat anti-mouse IgG conjugated with Cy2 (1:400, Jackson ImmunoResearch, West Grove, PA) for 2 h at room temperature. Control experiments included substitution of normal mouse serum for the primary antiserum and omission of the primary antiserum. Finally, the sections were rinsed in 0.01 M PBS and mounted onto gelatin-coated glass slides. Cover slips were applied with a mixture of 50% glycerin and 2.5% triethylene diamine in 0.01 M PBS.

Double-label immunofluorescence histochemistry was carried out as described previously [28,29]. Five sets of sections were incubated overnight at 4°C with primary mouse monoclonal anti-Kv1.2 and one each of the following primary antibodies: rabbit anti-NF200 (Sigma, St. Louis, MO), rabbit anti-P2X3 (Neuromics, Edina, MN), biotinylated IB4 (1:100, Sigma), rabbit anti- substance P (SP; Millipore), and rabbit anti- calcitonin gene-related peptide (CGRP; EMD, Gibbstown, NJ). The sections were then incubated for 1 h at 37°C with a mixture of goat anti-mouse IgG conjugated with Cy3 (1:400, Jackson ImmunoResearch) and donkey anti-rabbit IgG conjugated with Cy2 (1:400, Jackson ImmunoResearch) or with a mixture of goat anti-mouse IgG conjugated with Cy3 (1:400) and FITC-labeled avidin D (1:200, Sigma) for 1 h at 37°C. Control experiments as described above were performed in parallel. After the sections were rinsed in 0.01 M PBS, cover slips were applied as described above.

All immunofluorescence-labeled images were examined under a Nikon TE2000E fluorescence microscope (Nikon Co., Japan) and captured with a CCD spot camera. For single labeling, all labeled and unlabeled neurons with nuclei were counted. Cell profiles were outlined and cell area was calculated by using the imaging software Image-Pro Plus (Media Cybernetics, Silver Spring, MD). For double labeling, single-labeled and double-labeled neurons with nuclei were counted.

### Whole-cell patch clamp recording

Kv1.2 current was recorded in HEK293 cells transfected with Kv1.2 or EGFP plasmid by using whole-cell patch clamp recording 3–4 days after transfection. Cover slips were placed into the perfusion chamber (Warner Instruments, Hamden, CT) positioned on the stage of a microscope (Nikon); Micropipettes were pulled from borosilicate glass with a micropipette puller (PP-830, Narishige, Japan), and the electrode resistances ranged from 2 to 4 MΩ. Cells were voltage-clamped via the whole-cell configuration of the patch clamp with an Axopatch-700B amplifier (Molecular Devices, Sunnyvale, CA). The intracellular pipette solution (ICS) contained (in mM): potassium gluconate 120, KCl 20, MgCl_2_ 2, EGTA 10, HEPES 10, Mg-ATP 4 (pH = 7.3 with KOH, 310 mOsm). We minimized the Na^+^ and Ca^2+^ component in Kv current recording by using an extracellular solution composed of (in mM): choline-Cl 150, KCl 5, CdCl_2_ 1, CaCl_2_ 2, MgCl_2_ 1, HEPES 10, glucose 10 (pH = 7.4 with Tris-base, 320 mOsm). Signals were filtered at 1 kHz and digitized by using a DigiData 1322A with pClamp 9.2 software (Molecular Devices). Series resistance was compensated by 60–80%. Cell membrane capacitances were acquired by reading the value for whole-cell capacitance compensation directly from the amplifier. An online P*/*4 leak subtraction was performed to eliminate leak current contribution*.* The data were stored on computer by a DigiData 1322A interface and were analyzed by the pCLAMP 9.2 software package (Molecular Devices).

### Statistical analysis

The results from the behavioral tests, Western blot, and immunohistochemistry were statistically analyzed with a one-way or two-way analysis of variance (ANOVA). Data are presented as means ± SEM. When ANOVA showed a significant difference, pairwise comparisons between means were tested by the post hoc Tukey method. Significance was set at *p* < 0.05. The statistical software package SigmaStat (Systat, San Jose, CA) or GraphPad Prism 4.0 (GraphPad Software Inc., La Jolla, CA) was used to perform all statistical analyses.

## Results

### Distribution of Kv1.2-positive neurons in DRG

**Figure 1:**
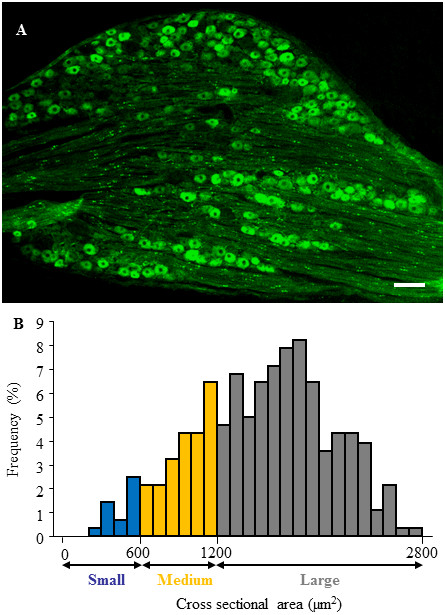
**Kv1.2 expression predominantly in the large and medium DRG neurons of normal rats. (A)** A representative image from immunohistochemical staining showing the distribution of Kv1.2-positive neurons. Scale bar: 200 μm. **(B)** Histogram showing the distribution of Kv1.2-positive somata.

**Figure 2:**
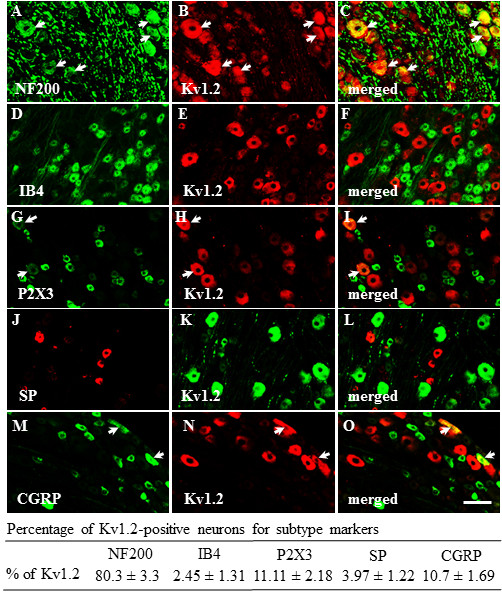
**Co-localization of Kv1.2 with NF200, IB4, P2X3, SP, and CGRP in DRG neurons.** Double immunohistochemical staining shows that approximately 80.3% of Kv1.2-labeled neurons are positive for NF200 **(A–C)**, 2.45% for IB4 **(D–F)**, 11.11% for P2X3 **(G–I)**, 3.97% for SP **(J–L)**, and 10.7% for CGRP **(M–O)**. Arrows: double-labeled neurons. Scale bar: 70 μm.

Consistent with previous reports [14], a majority (70%) of the DRG neurons are positive for Kv1.2 (Figure 1). Most of these positive neurons are medium and large in size. To further identify the cytochemical characteristics of Kv1.2-labeled neurons in the L_5_ DRG, we carried out double labeling immunohistochemistry for Kv1.2 and neuronal subtype markers (n = 5 rats). In neuron profiles, approximately 80.3 ± 3.3% of Kv1.2-labeled neurons were positive for NF200 (a marker for large cells and myelinated A-fibers; Figure 2), whereas approximately 11.11 ± 2.18% of Kv1.2-labeled neurons were positive for P2X3 (a marker for small DRG non-peptidergic neurons, Figure 2) and 10.7 ± 1.69% were positive for CGRP (a marker for small DRG peptidergic neurons; Figure 2). Interestingly, about 2.45 ± 1.31% of Kv1.2-labeled neurons were positive for IB4 (another marker for small DRG non-peptidergic neurons; Figure 2) and 3.97 ± 1.22% were positive for SP (another marker for small DRG peptidergic neurons; Figure 2).

### Reduction in number of Kv1.2-positive neurons in the injured DRGs after peripheral nerve injury

**Figure 3:**
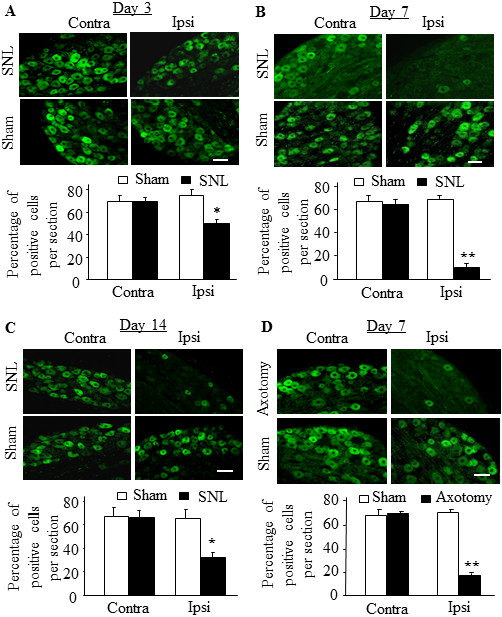
**Time-dependent decreases in the number of Kv1.2-positive neurons in the injured DRG after peripheral nerve injury.** Representative Kv1.2 immunohistochemical staining (Top) and statistical summary (Bottom) of the percentage of Kv1.2-positive neurons in the contralateral (Contra) and ipsilateral (Ipsi) L_5_ DRGs on days 3 **(A)**, 7 **(B)**, and 14 **(C)** after L_5_ spinal nerve ligation (SNL) or sham surgery and on day 7 after axotomy or sham surgery **(D)**. Scale bar: 100 μm. * *P* < 0.05, ** *P* < 0.01 compared to the number of Kv1.2-positive neurons on the contralateral side of sham-operated rats.

We next examined whether peripheral nerve injury altered number of Kv1.2-positive neurons in the injured DRG. Nerve injury caused by SNL (n = 4 rats/time point), but not sham surgery (n = 4 rats/time point), time-dependently decreased the number of Kv1.2-positive neurons in the ipsilateral L_5_ DRG (Figure 3A-C). The number of Kv1.2-positive neurons in the ipsilateral L_5_ DRG was significantly decreased by 25.2% on day 3 (*p* < 0.05), 84.8% on day 7 (*p* < 0.01) and by 52.2% on day 14 (*p* < 0.01) post-SNL compared to the number at the corresponding time points in the contralateral DRGs of the sham groups (Figure 3A-3C). These decreases occurred predominantly in large and medium DRG neurons. As expected, the number of Kv1.2-positive neurons in the contralateral L_5_ DRG did not change significantly at any time point post-SNL (Figure 3A-C). No dramatic changes in number of Kv1.2-positive neurons were also seen in either the ipsilateral or contralateral L_4_ DRG during the observation period (data not shown).

To further verify the effect of SNL on the number of Kv1.2-positive neurons in the injured DRG, we examined Kv1.2 immunostaining in the ipsilateral L_5_ DRG on day 7 after sciatic nerve axotomy or sham surgery. As expected, sham surgery did not significantly change the number of Kv1.2-psotive neurons in the ipsilateral or contralateral L_5_ DRG (Figure 3D). However, as in the SNL model, the number of Kv1.2-positive neurons in the ipsilateral L_5_ DRG was reduced by 75.6% (*p* < 0.01; n = 4 rats) after axotomy compared to that after sham surgery (n = 4 rats) (Figure 3D).

### Over-expressing Kv1.2 in the injured DRG blunted neuropathic pain without affecting basal nociceptive responses

**Figure 4:**
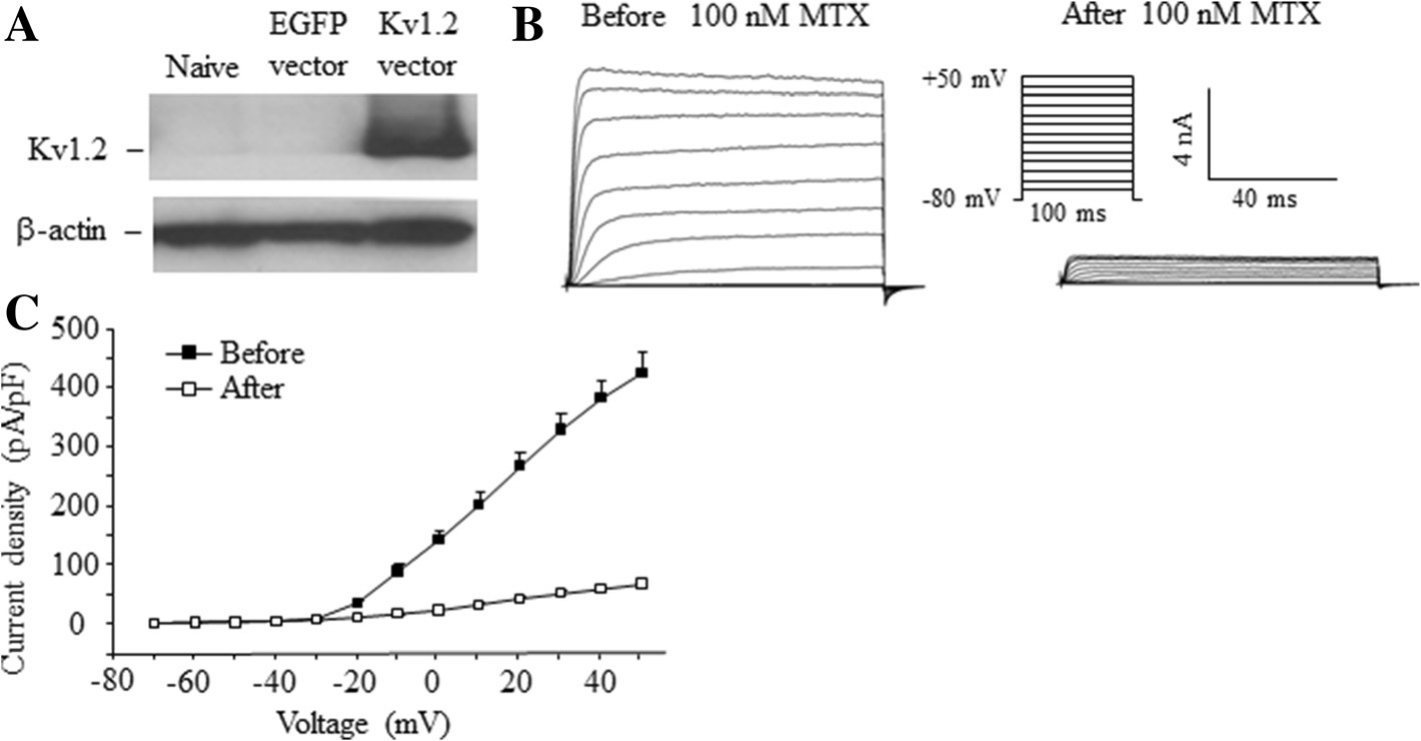
***In vitro *expression and current of Kv1.2 in HEK-293 cells transfected with Kv1.2 vector. (A)** Representative Western blot showing Kv1.2 expression at the expected size in HEK-293 cells transfected with full-length Kv1.2 vector. β-actin was used as a loading control. **(B)** Representative traces of Kv1.2 current recorded by whole-cell voltage patch clamp in HEK-293 cells transfected with full-length Kv1.2 vector before or after bath perfusion of 100 nM maurotoxin (MTX). **(C)** Current–voltage curves of HEK-293 cells transfected with full-length Kv1.2 before or after treatment with 100 nM MTX. The current density was plotted against each step testing voltage. n = 5 cells.

Kv1.2 controls neuronal excitability [30]. Peripheral nerve injury-induced Kv1.2 downregulation might engage in abnormal spontaneous activity in the injured DRG neurons and participate in the mechanisms that underlie neuropathic pain [16]. Thus, rescuing Kv1.2 downregulation in the injured DRG may affect nerve injury-induced pain hypersensitivity. To this end, a full-length Kv1.2 cDNA was cloned into a proviral AAV5 vector to replace EGFP cDNA. As shown in Figure 4A, cultured HEK-293 cells transfected with Kv1.2 vector, but not EGFP vector, expressed Kv1.2 protein at the expected size. To further test whether this cloned protein could produce functional currents, we carried out voltage-clamped recording. As expected, no voltage-dependent current was detected in the EGFP vector-transfected cells (data not shown). However, Kv1.2 vector-transfected cells exhibited voltage-dependent currents (Figure 4B). These current densities could be blocked dramatically by a selective Kv1.2 current inhibitor, maurotoxin [31-33] (Figure 4B and 4C), indicating that the recording currents are Kv1.2-mediated.

**Figure 5:**
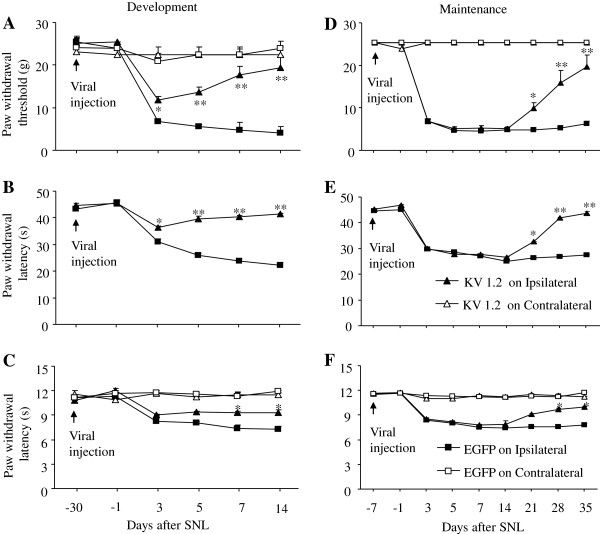
**Overexpressing Kv1.2 in the injured DRG mitigates neuropathic pain.** Kv1.2 was delivered into the injured DRG through microinjection of AAV5-Kv1.2. AAV5-EGFP was used as a control. **(A-C)** Behavioral tests show the effect of overexpressing Kv1.2 on the development of spinal nerve ligation (SNL)-induced pain hypersensitivities. Ipsilateral and contralateral paw withdrawal responses to mechanical **(A)**, cold **(B)**, and thermal **(C)** stimuli at the times shown before and after SNL. n = 5/group. *P < 0.05, **P < 0.01 vs. the ipsilateral side of the AAV5-EGFP-treated group at the corresponding time point. **(D-F)** Behavioral tests show the effect of overexpressing Kv1.2 on the maintenance of SNL-induced pain hypersensitivities. Ipsilateral and contralateral paw withdrawal responses to mechanical **(D)**, cold **(E)**, and thermal **(F)** stimuli at the times shown before and after SNL. n = 5/group. *P < 0.05, **P < 0.01 vs. the ipsilateral side of the AAV5-EGFP-treated group at the corresponding time point.

To overexpress Kv1.2 RNA in DRG *in vivo*, we injected AAV5-Kv1.2 viral particles into the left L5 DRG. The same amount of AAV5-EGFP viral particles was injected as a control. Consistent with the previous reports [16,18,19], the injected rats showed no signs of paresis or other abnormalities. The injected DRGs, stained with hematoxylin/eosin, retained their structural integrity and contained no visible leukocytes (data not shown) indicating that the immune responses from viral injection were minimal. Because AAV5 takes 3–4 weeks to become expressed in *in vivo*[16,18,19], SNL was carried out 4 weeks after injection. We first compared paw withdrawal responses to mechanical, cold, or thermal stimuli between pre- and post-viral injection. There were no significant changes in paw withdrawal thresholds or latencies on either hind paw 4 weeks after viral injection compared to the baseline thresholds or latencies (Figure 5). We then examined whether over-expressing Kv1.2 RNA in the injured DRG affected neuropathic pain development. As expected, SNL produced mechanical, cold, and thermal hypersensitivities on the ipsilateral side in the EGFP-injected group (n = 5 rats; Figure 5A-C). However, hypersensitivity was attenuated in the Kv1.2-injected rats (n = 5 rats; Figure 5A-C). Paw withdrawal threshold to mechanical stimulation and paw withdrawal latencies to cold and thermal stimuli were higher in the Kv1.2-injected rats than in the EGFP-injected group from day 3 to day 14 post-SNL (Figure 5A-C). To further investigate the effect of over-expressing Kv1.2 RNA on neuropathic pain maintenance, we subjected rats to SNL one week after DRG viral injection. As shown in Figure 5D-F (n = 5 rats/group), mechanical, cold, and thermal hypersensitivities completely developed in both the Kv1.2-injected and EGFP-injected rats from day 3 to day 14 post-SNL (Figure 5D-F). These hypersensitivities were attenuated on days 21, 28, and 35 post-SNL in the Kv1.2-injected rats compared to the EGFP-injected rats (Figure 5D-F). Neither AAV5-Kv1.2 nor AAV5-EGFP affected paw withdrawal threshold or latency on the contralateral side in the development and maintenance of SNL-induced neuropathic pain (Figure 5).

### Over-expressing Kv1.2 in the DRG did not alter capsaicin-induced acute pain

**Figure 6:**
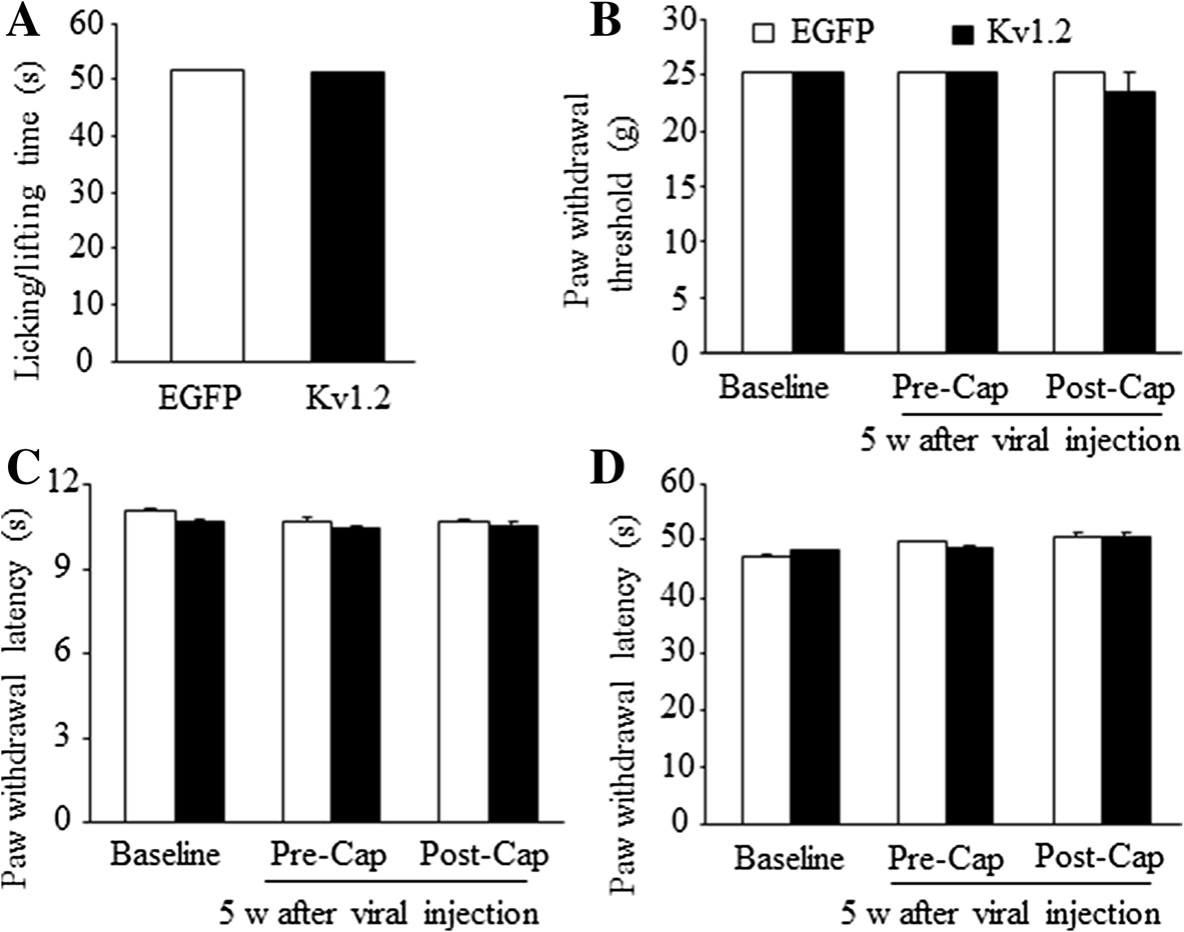
**Overexpressing Kv1.2 in the DRG does not alter capsaicin-induced acute nociceptive pain. (A)** The duration of capsaicin-induced paw licking/lifting was recorded for 5 minutes after capsaicin injection in the AAV5-EGFP-injected and AAV5-Kv1.2-injected groups. n = 5/group. **(B-D)** Paw withdrawal responses of AAV5-EGFP-injected and AAV5-Kv1.2-injected rats to mechanical **(B)**, heat **(C)**, and cold **(D)** stimuli on the ipsilateral side prior to viral injection (baseline), before capsaicin injection (Pre-Cap; 5 weeks after viral injection), and 30 minutes after capsaicin injection (Post-Cap). n = 5/group.

To further examine whether over-expressing Kv1.2 in the DRG altered acute pain, we subjected rats to capsaicin and observed capsaicin-induced acute nociceptive pain characterized by licking and/or lifting of the injected hind paw (n = 5 rats/group). Consistent with the previous reports [24], the nocifensive response evoked by capsaicin occurred predominantly during the first 5-min observation period. We found that capsaicin-induced the paw licking/lifting response in this period from the Kv1.2-injected group was similar to that from the EGFP-injected group (Figure 6A). We further tested paw withdrawal responses to mechanical, thermal, and cold stimuli 30 min after capsaicin injection. No significant differences were observed between the EGFP-injected and Kv1.2-injected groups (Figure 6B and 6C).

### Over-expressing Kv1.2 RNA in the DRG did not affect locomotor functions

We also determined whether over-expressing Kv1.2 RNA influenced locomotor function of experimental animals. As shown in Table 1, neither AAV5-Kv1.2 nor AAV5-EGFP produced any effect on locomotor functions including placing, grasping and righting reflexes in naïve (n = 5), EGFP-treated (n = 5), and Kv1.2-treated (n = 5) rats. Convulsions and hypermobility were not observed in any of the treated animals. In addition, we did not observe any significant difference in general behaviors, including the gait and spontaneous activity, between naïve rats and the AAV5-treated groups.

**Table 1:** Mean (± SDM) changes in locomotor test

**Groups**	**Placing**	**Grasping**	**Righting**
EGFP in naive	5 (0)	5 (0)	5 (0)
Kv1.2 in naive	5 (0)	5 (0)	5 (0)
EGFP in sham	5 (0)	5 (0)	5 (0)
Kv1.2 in sham	5 (0)	5 (0)	5 (0)
EGFP in SNL	5 (0)	5 (0)	5 (0)
Kv1.2 in SNL	5 (0)	5 (0)	5 (0)

N = 5/group, 5 trials.

### Over-expressing Kv1.2 RNA in the DRG attenuated SNL-induced DRG Kv1.2 antisense (AS) RNA upregulation

**Figure 7:**
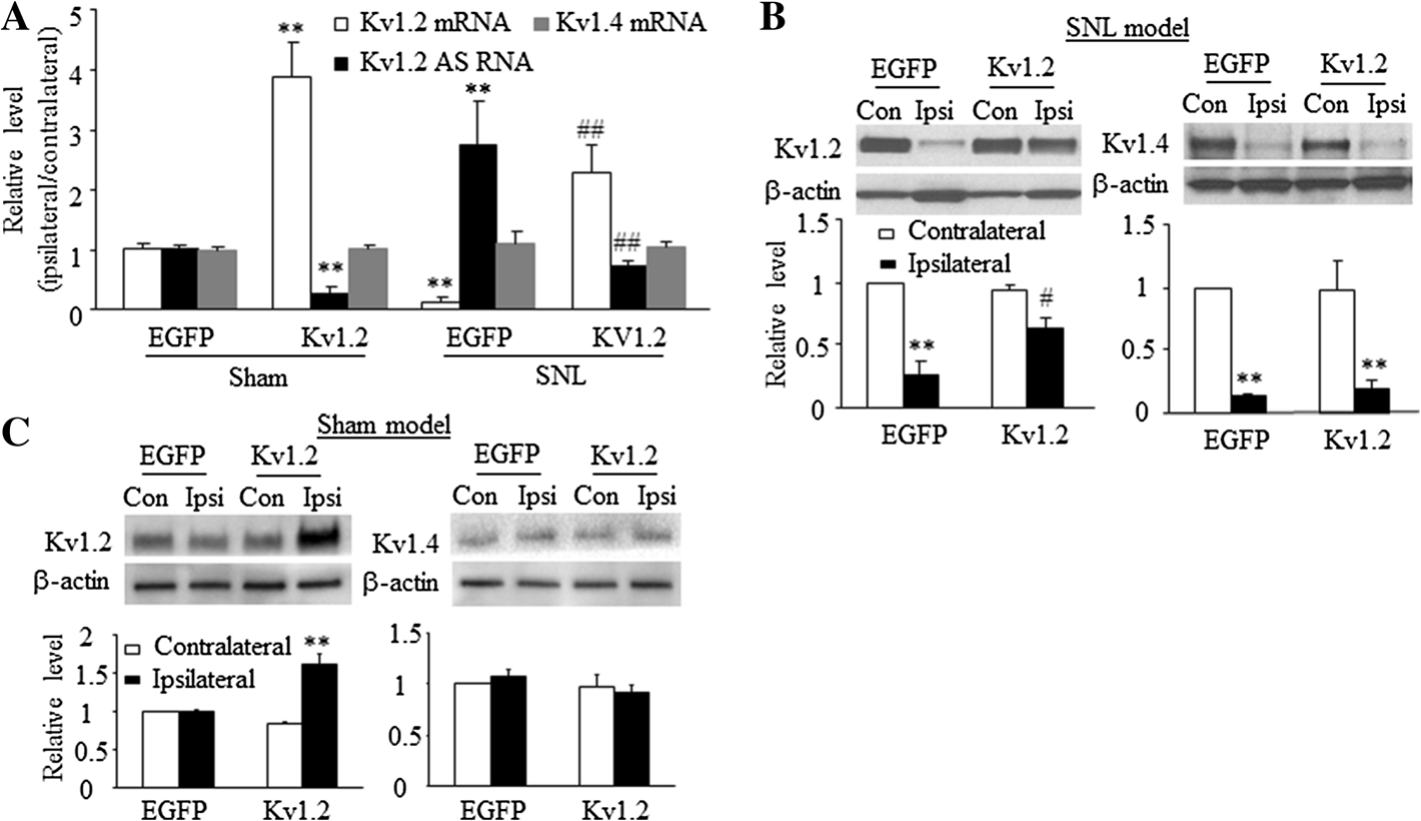
**Overexpressing Kv1.2 rescues SNL-induced Kv1.2 downregulation and blocks SNL-induced Kv1.2 AS RNA upregulation in the injured DRG. (A)** Quantitative RT-PCR shows Kv1.2 AS RNA, Kv1.2 mRNA, and Kv1.4 mRNA expression in the ipsilateral and contralateral L_5_ DRGs on day 14 after spinal nerve ligation (SNL) or sham surgery in the AAV5-EGFP-injected and AAV5-Kv1.2-injected groups. n = 12 rats/group. **P < 0.01 vs. the corresponding AAV5-EGFP-injected group after sham surgery; ##*P* < 0.01 vs. the corresponding AAV5-EGFP-injected group after SNL. **(B)** Western blot analysis shows Kv1.2 and Kv1.4 protein expression in the ipsilateral (Ipsi) and contralateral (Con) L_5_ DRGs on day 14 after SNL in the AAV5-EGFP-injected and AAV5-Kv1.2-injected groups. n = 8 rats/group. **P < 0.01 vs*.* the contralateral side of the AAV5-EGFP-injected group; #*P* < 0.05 vs*.* the ipsilateral side of the AAV5-EGFP-injected group. **(C)** Western blot analysis shows Kv1.2 and Kv1.4 protein expression in the ipsilateral and contralateral L_5_ DRGs on day 14 after sham surgery in the AAV5-EGFP-injected and AAV5-Kv1.2-injected groups. n = 8 rats/group. **P < 0.01 vs. the contralateral side of the AAV5-EGFP-injected group.

We recently reported that Kv1.2 AS RNA as a trigger contributes to neuropathic pain development and maintenance by specifically silencing Kv1.2 mRNA in the DRG [16]. Therefore, we finally defined whether the antinociceptive effect of DRG Kv1.2 RNA overexpression was attributed to the expressional change of DRG Kv1.2 AS RNA. Consistent with our previous work [16], SNL downregulated Kv1.2 mRNA and protein and upregulated Kv1.2 AS RNA in the injured DRG 14 days after SNL in the EGFP-injected group (n = 5 rats; Figure 7A and B). Over-expressing Kv1.2 RNA in the injured DRG not only rescued the SNL-induced decrease in DRG Kv1.2 mRNA and protein but also blocked the SNL-evoked increase in DRG Kv1.2 AS RNA in the Kv1.2-injected group (Figure 7A and B). In sham rats, over-expressing Kv1.2 RNA in the DRG also inhibited basal DRG Kv1.2 AS RNA expression in addition to increasing basal DRG Kv1.2 mRNA and protein (Figure 7A and C). The levels of Kv1.4 mRNA and protein were unaffected by the over-expression of DRG Kv1.2 RNA in either SNL or sham rats compared to the corresponding EGFP-injected group (Figure 7A-C).

## Discussion

Three major findings arise from the present study. First, Kv1.2 expressed in a majority of the large and medium DRG neurons is time-dependently downregulated in the injured DRG following peripheral nerve injury induced by L_5_ SNL and sciatic nerve axotomy. Second, rescuing this downregulation by over-expressing DRG Kv1.2 RNA blocked the development and maintenance of SNL-induced neuropathic pain. Finally, over-expressing DRG Kv1.2 did not affect basal acute pain, capsaicin-induced acute pain, and locomotor functions. These findings suggest that the DRG Kv1.2 channel may be a novel potential target for treating neuropathic pain.

Normal DRG expresses several Kv1 alpha subunits at various levels of basal expression. RT-PCR analysis showed that Kv1.1 and Kv1.2 mRNA was highly abundant, whereas Kv1.3, Kv1.4, Kv1.5, and Kv1.6 mRNA was present at lower levels [12]. Immunohistochemistry further revealed that Kv1.1, Kv1.2, and Kv1.4 protein was detected highly in DRG neurons [14]. In contrast, Kv1.3, Kv1.5, and Kv1.6 protein expression was very low or undetectable in the DRG [14]. Consistent with these findings, we found that approximately 70% of DRG neurons expressed Kv1.2 protein in neuron profiles. These findings suggest that Kv1.2, Kv1.1, and Kv1.4 are key subunits in formation of heteromeric Kv channels in DRG neurons.

Kv1 alpha subunits are present in distinct classes of DRG neurons. An early study reported that Kv1.1 and Kv1.2 are expressed in small-sized DRG neurons [13]. This finding was not confirmed in a subsequent study that showed Kv1.2 and Kv1.1 to be expressed predominantly in medium- and large-sized DRG neurons [14]. We used specific cytochemical markers and the measurement of neuronal cell body area to further characterize Kv1.2 expression in functional classes of DRG neurons. In neuron profiles, approximately 72% of Kv1.2-positive neurons were large, 19% were medium, and 9% were small. Consistently, we found that most (80.3%) Kv1.2 co-localized with NF200, a marker for large, myelinated afferents, and some co-localized with P2X3 (11.11%) and CGRP (10.7%), markers of small nociceptive cells. Unexpectedly, few Kv1.2-positive neurons co-expressed two other markers of small nociceptive cells, IB4 (2.45%) and SP (3.97%). It is unclear why Kv1.2 showed different levels of co-localization with distinct nociceptive markers, but the observation may be related to different subpopulation distribution of these markers in rat DRG. CGRP and P2X3 are expressed not only in small DRG neurons but also in medium and/or large DRG neurons, whereas SP and IB4 are expressed exclusively in small DRG neurons [34-36]. In addition, CGRP is co-expressed with P2X3, but not with IB4, in some DRG neurons [37]. Unique subpopulation distribution of Kv1.2 in DRG suggests a functional consequence of its down-regulation on pain-associated behaviors following peripheral nerve injury.

Nerve injury-induced Kv1.2 downregulation in the injured DRG may participate in neuropathic pain development and maintenance. Data from the present study and those of others [12-16,38,39] showed a time-dependent decrease in expression of Kv1.2 mRNA and protein in the injured DRG neurons following peripheral nerve injury. This decrease occurred predominantly in large and medium DRG neurons. We recently demonstrated that DRG Kv1.2 reduction in the large and medium DRG neurons decreased total voltage-gated potassium current, depolarized the resting membrane potential, decreased current threshold for activation of action potentials, increased the number of action potentials in these DRG neurons, and produced neuropathic pain symptoms [16]. Blocking SNL-induced reduction of Kv1.2 expression in the injured DRG attenuated neuropathic pain during development and maintenance periods [16]. The evidence indicates that DRG Kv1.2 is a key player in neuropathic pain genesis and may be a potential target for preventing and/or treating neuropathic pain. Consistent with this speculation, the present study demonstrated that rescuing Kv1.2 expression in the injured DRG diminished the induction and maintenance of SNL-induced mechanical, cold, and thermal pain hypersensitivities. These behavioral effects may be attributed to direct compensation of SNL-induced reduction of Kv1.2 protein and inhibition of SNL-induced upregulation of Kv1.2 AS RNA in the injured DRG. The latter may be related to the extensive overlap of complimentary regions between Kv1.2 mRNA and Kv1.2 AS RNA [16]. Given that the nerve injury-induced increase in spontaneous ectopic activity in the injured myelinated afferents [40-42] is believed to play a leading role in the genesis of neuropathic pain [1,43], rescuing Kv1.2 downregulation may maintain normal resting membrane potential and reduce abnormal ectopic activity in the injured DRG neurons. This effect may decrease primary afferent transmitter release and result in attenuation of spinal central sensitization formation and nerve injury-induced pain hypersensitivity. Interestingly, rescuing DRG Kv1.2 expression does not alter acute pain, which is supported by the observation that the altered DRG Kv1.2 expression did not change the threshold of action potential [16]. Therefore, the strategy of rescuing DRG Kv1.2 expression may be a novel treatment for neuropathic pain.

## Competing interests

The authors declare that they have no competing financial interests.

## Authors’ contributions

YXT conceived and designed the project and supervised all experiments. LF and VT performed animal models and behavioral tests. XG and WW performed immunohistochemical experiments. JYZ and LF performed in vitro transfection and quantitive RT-PCR experiments. HZ and ML carried out voltage clamp experiments. PNH did microinjection. LF, XG, WW, HZ, VT, ML, and YXT analyzed the data. YXT wrote the manuscript. All of the authors read and approved the manuscript.
